# Extraperitoneal laparoscopic radical cystectomy with intracorporeal neobladder: a comparison with transperitoneal approach

**DOI:** 10.1186/s12957-022-02587-1

**Published:** 2022-04-23

**Authors:** Ying Zhang, Huan Zhou, Zhou Ting Tuo, Jinyou Wang, Chenyu Sun, Liangkuan Bi

**Affiliations:** 1 Department of Urology, The Second Affiliated Hospital of Anhui Medical University, 668 Furong Road, Hefei, 230032 Anhui China; 2 Internal Department, AMITA Health Saint Joseph Hospital, 2900 N. Lake Shore Drive, Chicago, IL 60657 USA

**Keywords:** Laparoscopic radical cystectomy, Extraperitoneal, Transperitoneal, Surgical technique, Urinary diversion, Bladder cancer, Intracorporeal urinary diversion, Laparoscopy, Radical cystectomy

## Abstract

**Background:**

Bladder cancer is one of the most common genitourinary cancers. Traditional transperitoneal radical cystectomy is the gold standard treatment for muscle-invasive bladder cancer. Our study was to compare the perioperative and oncological outcomes of extraperitoneal laparoscopic radical cystectomy (ELRC) with intracorporeal neobladder versus transperitoneal urinary diversion for bladder cancer.

**Method:**

A total of 113 patients who underwent laparoscopic radical cystectomy performed at our center were included in this retrospective study. The perioperative data of the extraperitoneal laparoscopic radical cystectomy (ELRC) with intracorporeal urinary diversion (ICUD) and transperitoneal laparoscopic radical cystectomy (TLRC) with ICUD groups were compared. The demographic, perioperative, oncological, and complication data were collected and analyzed.

**Results:**

In total, 113 patients were enrolled for the final analysis. The median follow-up period was 22 months. The ELRC group had shorter interval to flatus (*p* < 0.001), solid food (*p* < 0.001), shorter length of hospital stay (*p* < 0.01), and fewer early gastrointestinal complications (*p* < 0.05). Furthermore, urinary continence, recurrence-free, cancer-specific, and overall survival rates and recurrence patterns did not significantly differ.

**Conclusions:**

Surgical technique of ELRC with ICUD can achieve the established oncologic criteria of TLRC, and such technique can improve perioperative and early postoperative outcomes.

## Background

For decades, radical cystectomy with urinary diversion has been the standard treatment for non-metastatic muscle-invasive and high-risk non-muscle-invasive bladder cancer [1]. Due to needs of spontaneous voiding and quality of life, continent urinary diversion to the intact urethra, such as ileal neobladder, has become mainstream in tertiary institutions, which is performed in over 50% patients [2]. The transperitoneal approach is currently the most commonly used method, which involves transperitoneal antegrade mobilization of the bladder with blunt dissection [3]. The transperitoneal route destroys the original physiological membrane structure and increases the exposure of the bowels. This may explain the high complication rates ranging from 40–44%, even with the assistance of robotic systems [4, 5]. In 1999, Kulkari et al. first reported their extraperitoneal approach with ideal outcomes [3], and satisfactory functional and oncological outcomes were revealed by Kulkarni et al. in 2018 [6]. In our study, laparoscopic extraperitoneal radical cystectomy and laparoscopic transperitoneal approach were compared to investigate the morbidity and histopathologic outcomes. The center is experienced with laparoscopic radical cystectomy and urinary diversion in the region of Anhui Province. From 2014, our center adopted laparoscopic radical cystectomy (LRC) with intracorporeal ileal neobladder as a standard treatment for muscle invasive bladder cancer (MIBC). In 2018, the approach of extraperitoneal laparoscopic radical cystectomy (ELRC) with ileal neobladder was explored. Surgeons performing ELRC were required to perform over 40 cases which were similar to experiences described by Justin W et al. [7].

## Methods

### Surgical technique

#### Preoperative preparation

The patients had liquid diet for 2 days prior to the surgery. Following the enhanced recovery after surgery (ERAS) protocol, nasogastric tube and fully bowel preparation were not used.

### Surgical technique of ELRC

After general anesthesia, all the patients were placed in the Trendelenburg position to create more room for operation. Surgical steps of ELRC could be watched by the link of https://youtu.be/9tGn-jv5tWU.

**Fig. 1:**
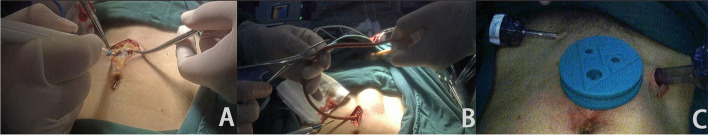
**A** 4 cm infraumbilical skin incision to enter the extraperitoneal space. **B** An artificial gasbag was inserted and injected with air inflation of 700–1000 ml to expand the extraperitoneal operation space. **C** The trocars (12 mm and 5 mm) location

**Fig. 2:**
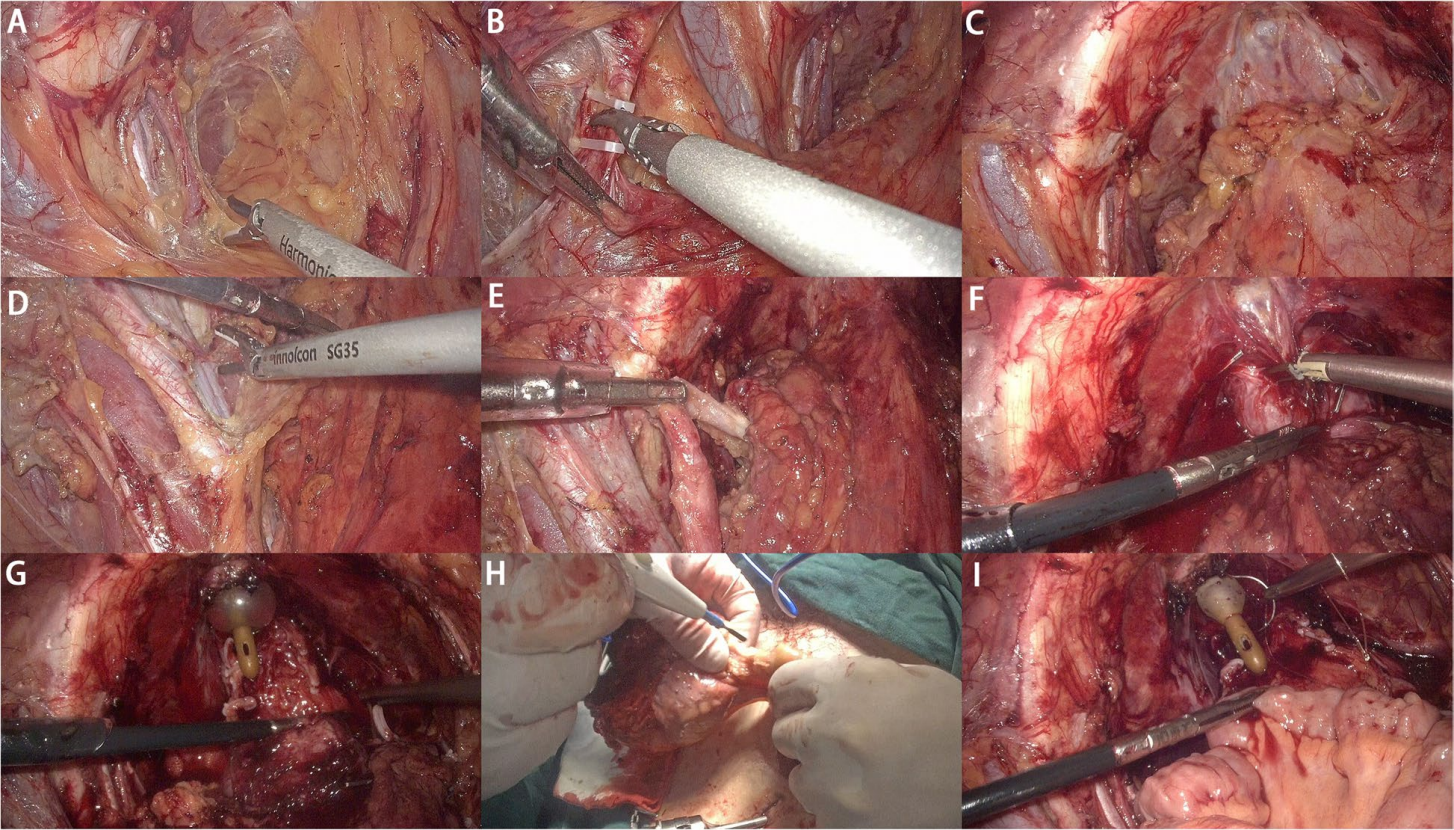
**A** Whole pelvic fatty tissue was gently pushed with ultrasonic knife. **B** Vas deferens were cut off. **C** The left space of the bladder was fully freed. **D** PLND of the left side. **E** Cutting off the left ureter. **F** Resection of urethra and prostate. **G** Pelvic view after RC. **H** Resecting the bladder in vitro. **I** The beginning of reconstruction of intracorporeal ileal neobladder

First, extraperitoneal space was established. A 4-cm infraumbilical skin incision to enter the extraperitoneal space (Fig. 1A). Then, an artificial gasbag was inserted and injected with air inflation of 700–1000ml to expand the extraperitoneal operation space of Retzius (Fig. 1B). The trocars (diameter 12 mm and 5 mm) of the second and third puncture points were then placed with guidance of fingers, located along the pararectal line at 4 cm (left side) and 2 cm (right side) inferior to the umbilicus level, respectively (Fig. 1C). After closing the abdominal wall, we inserted the first trocar. The second step was radical cystectomy and bilateral pelvic lymph node dissection (PLND). When the space of Retzius was entered, operability was assessed by palpating the bladder tumor and its mobility in the pelvis. The whole pelvic fat tissue was gently pushed with ultrasonic knife (Fig. 2A). After exposing the vas deferens on both sides, the vas deferens were cut off (Fig.2B) and the lymph node dissection (Fig. 2D) was performed at the level of the iliac artery. At the same time, left space of the bladder was shown (Fig. 2C). The hypogastric artery was divided between ligatures at its origin from the internal iliac artery. The urachus was cut at the level of the umbilicus. The ureter on left side was identified and mobilized to the ureterovesical junction (Fig. 2E) [8]. Prostatic adipose tissue was teased away to expose the puboprostatic ligaments and dorsal venous complex (Fig.2F). The puboprostatic ligaments and prostatic lateral ligament were divided and clamped with Hem-o-lok. The anterior urethral wall was incised along the striated sphincter to expose the urethral catheter (Fig. 2G). Then the posterior urethral wall was incised distal to expose Denonvilliers’ fascia. During the removal of the prostate, the pudendal nerves and blood vessels were fully preserved. The last step of ELRC was resecting the bladder in vitro (Fig. 2H). Then the intracorporeal neobladder construction steps were begun (Fig. 2I).

### Surgical technique of TLRC

The approach of TLRC was performed according to the techniques described by Huang et al. [9]

The intracorporeal neobladder technique of urinary diversion was described by Lu et al. in 2020 [10] and Zhang et al. in 2021 [11]. Video link is https://youtu.be/JyT_-Aa3dL4.

### Postoperative treatment

All patients who underwent LRC were monitored in intensive care unit (ICU) on the first postoperative day. After passage of flatus, patients were started with up to 100ml of water or juice. Then they were transferred from ICU to urology ward and gradually advanced from a liquid diet to a solid food during the first week.

### Follow-up

After institutional review board approval (AHMU-1876), the data were retrieved from the prospectively owned database of a tertiary referral medical center in Anhui Province, China. Informed consent was obtained from all of the patients or their guardians. The inclusion criteria were muscle-invasive or recurrent high-risk bladder tumors that were non-responsive to intravesical immunotherapy. The clinical stage was cT1-T3. The contraindications included (1) severe obesity (body mass index BMI ≥ 35 kg/m^2^); (2) distant metastases; (3) poor renal function; (4) severe liver insufficiency; (5) active enteritis; (6) severe cardiopulmonary dysfunction, and (7) positive urethral margins. From January 2018 to December 2019, a total of 139 patients received neobladder urinary diversion. Of these patients, 113 met the eligibility criteria for inclusion in our study. All patients assigned the informed consent before enrollment to the study. Thirty-eight patients underwent ELRC with intracorporeal neobladder, while 65 patients underwent transperitoneal laparoscopic radical cystectomy (TLRC) with intracorporeal neobladder.

All data were retrospectively collected based on medical records. The mean follow-up was 15 months. Major complications were defined as grade 3–5 of Clavien-Dindo systems [12]. Complications were classified as early (≤ 30 days postoperatively) and late (> 30 days postoperatively). Ileus and renal failure were common complications during perioperative period. Ileus was defined with as persistent abdominal pain after surgery and no stool until postoperative day 7. Renal function was estimated usually with creatinine and ultrasound scanning postoperatively. The cause of acute renal insufficiency (AKI) after LRC was often related to anastomotic leakage. Daytime or nocturnal continence was defined as the use of ≤ 1 pad, while incontinence was defined as the use of > 1 pad.

Statistical calculations were performed with SPSS 22.0 (Chicago, USA). Analysis was done according to data scaling using the unpaired Student t test and the chi-square test or, for lower expectancy rates, Fisher’s exact test. When the *p* value was below 0.05, data were considered significantly different.

## Results

Of the 113 patients, 48 underwent ELRC with intracorporeal neobladder and 65 patients underwent TLRC. The baseline demographic data are shown in Table 1. There was no statistically significant difference between ELRC and TLRC for age (67.8 ± 8.4 vs 66.0 ± 8.9 years; *p* = 0.283), BMI (25.5 ± 3.6 vs 26.2 ± 3.6; *p* = 0.313). All patients were men. In terms of American Society of Anesthesiologists (ASA) score distribution and previous abdominal surgery history, there were no significant differences. As for clinical stage distribution, new adjuvant chemotherapy (NAC) and transurethral resection of bladder tumor (TUR-BT) history, such data were comparable without significant difference.

**Table 1:** Baseline and perioperative demographic data

Characteristic	ELRC group (*n* = 48)	TLRC group (*n* = 65)	*p* value
Age year, median ± SD (range)	67.8 ± 8.4	66.0 ± 8.9	0.283^c^
BMI kg/m^2^, median ± SD (range)	25.5 ± 3.6	26.2 ± 3.6	0.313^c^
ASA score ≥ 2, no. (%)	25(52.1)	38(58.5)	0.567^a^
Previous abdominal surgery, no. (%)	13 (27.1)	20 (30.8)	0.835^a^
**Preoperative clinical stage**			0.563^a^
Ta Tis	5 (10.4)	9 (13.8)	
T1	16 (33.3)	26 (40.0)	
T2	27 (56.3)	30 (46.2)	
TUR-BT, *n* (%)	23 (47.9)	38 (58.5)	0.340^a^
New adjuvant chemotherapy, *n* (%)	14 (29.2)	21 (32.3)	0.837^a^
Operative time, median ± SD, min	286.5 ± 34.5	272.1 ± 35.5	**0.036**^**b**^
Estimated blood loss, mean ± SD, mL	405.2 ± 173.3	383.0 ± 180.0	0.517^c^
Transfusion rate, no. (%)	3(6.3)	5(7.7)	0.999^b^
Interval to flatus, mean ± SD, h	35.5 ± 9.7	42.7 ± 10.8	**0.0004**^**c**^
Interval to solid food, mean ± SD, day	4.8 ± 1.3	6.0 ± 1.7	**0.0017**^**c**^
Length of hospital stay, mean± SD, day	12.7 ± 2.0	14.0 ± 2.7	**0.0049**^**c**^

^a^Pearson chi-square test

^b^Fisher exact test

^c^Independent *t* test

### Perioperative parameters

The mean operative time was longer in the ELRC group vs TLRC group (286.5 ± 34.5 vs 272.1 ± 35.5, *P* = 0.036). There was no significant difference between the two groups in terms of EBL (*p* = 0.517). Transfusion rate (*p* = 0.999). The interval to flatus (*p* < 0.001), interval to solid food (*p* < 0.01), and length of hospital stay (LOS) (*p* < 0.01), which were used as indicators of the bowel recovery time, were significantly shorter in ELRC group (Table 1).

### Postoperative outcomes and complications

Early and late complications were shown in Tables 2 and 3 respectively. Within 30 days postoperatively, one patient in the TLRC group suffered severe ileus, intestinal fistula, and peritonitis. He immediately received CT scanning and reoperation, and eventually recovered. During surgery, it was found that anastomotic leakage was due to suture rupture. For the other patient, the intestinal obstruction was caused by the adhesion of the intestine to the peritoneum, which lead the intestinal lumen to be blocked.

**Table 2:** Comparison of early complications in ELRC group versus TLRC group

Early complications	ELRC group (*n* = 48)	TLRC group (n = 65)	*p* value
Over all complication rate (at least one)	15 (31.3%)	31 (47.7%)	0.0856^b^
Major complication (grade 3–5)	5 (10.4%)	9 (13.8%)	0.774^b^
**Gastrointestinal complications**	1 (2.1%)	10 (14.9%)	**0.0231**^**b**^
No stool until postoperative day 7	1	6	
Ulcer or gastrointestinal bleeding	0	3	
Intestinal fistula and peritonitis (reoperation)	0	1	
**Infection**	4 (8.3%)	7 (10.8%)	0.757^b^
Wound infection	2	4	
Pneumonia	2	3	
**Urinary complications**	4 (8.3%)	5 (7.7%)	0.999^b^
Urinary tract infection	1	2	
Urine leakage	1	1	
Pyelonephritis	0	1	
Renal failure	2	1	
**Wound dehiscence**	3 (6.3%)	2 (3.1%)	0.649^b^
**Bleeding postoperatively**	1 (2.1%)	3 (4.6%)	0.636^b^
**Lymphocele**	2 (4.2%)	2 (3.1%)	0.999^b^
**Cardiac dysfunction**	0 (0)	1 (1.5%)	0.999^b^
**Thromboembolic**	2 (4.2%)	3 (4.6%)	0.999^b^
Deep leg vein thrombosis	1	3	
Pulmonary embolism	1	0	
**Neurosystem complications**	0(0)	0(0)	0.999^b^

^a^Pearson chi-square test

^b^Fisher exact test

^c^Independent *t* test

**Table 3:** Comparison of late complications in ELRC group versus TLRC group

Late complications	ELRC group (*n* = 48)	TLRC group (*n* = 65)	*p* value
Over all complication rate (at least one)	13 (27.1%)	22 (33.8%)	0.538^b^
Major complications (grade 3–5)	5 (10.4%)	11 (16.9%)	0.418^b^
**Gastrointestinal**	**3 (6.3%)**	**8 (12.3%)**	0.349^b^
Conservative management	3	6	
Reoperation	0	2	
**Genitourinary**	**12 (25.0%)**	**21 (32.3%)**	0.531^b^
**Uretero-ileal stenosis**			
Conservative management	1	3	0.349^b^
Reoperation	2	5	
**Vesico-urethral anastomotic stricture**			0.999^b^
Conservative management	2	3	
Reoperation	0	0	
Urinary tract infection	4	7	
Neobladder stone	3	3	
**Metabolic acidosis**	2	1	0.574^b^
**Incisional hernia**	0	0	0.999^b^

According to Clavian-Dindo classification, 31.3% patients in the ELRC group suffered early complications compared with 47.7% patients in the TLRC group. Additionally, 27.1% patients in the ELRC group vs 33.8% patients in the TLRC group had experienced late complications in the follow-up, respectively. There was no significant difference between the two groups in terms of overall early or late complication rate. In the both groups, most common early complications were gastrointestinal, infectious, and urinary complications. The gastrointestinal complications were significantly less in the ELRC group (*p* = 0.023). Late complications were most urinary diversion-related, including uretero-ileal stenosis, vesical-urethral anastomotic stricture, pyelonephritis, and neobladder stone. Metabolic acidosis and incisional hernia were also reported. Complications of grade 3–5 were considered as major complications. Within the first 30 days, 5(10.4%) patients in the ELRC and 9 (13.8%) patients in the TLRC group had suffered from major complications. Late major complications occurred in 5(10.4%) of the ELRC group and 11(16.9%) of the TLRC group. No significant difference was found between the groups in late major complications.

### Pathologic outcomes

For those patients whose oncological data could be retrieved, the pathologic outcomes were comparable between the two groups (Table 4). The overall pathology stages were not significantly different (*p* = 0.793). Positive surgical margins were reported in 3 patients in the ELRC group and 5 patients in the TLRC group, without statistically significant difference (*p* > 0.999). There was no significant difference in terms of lymph node yield between the 2 groups (*p* = 0.638).

**Table 4:** Final pathology outcomes and oncological outcomes in ELRC group versus TLRC group

Outcomes	ELRC (***n*** = 48)	TLRC (***n*** = 65)	***p*** value
Local recurrence	1	3	0.636^b^
Distant metastasis	2	2	0.999^b^
Cancer-specific mortality for 2 years	2	3	0.999^b^
Non-cancer-specific mortality for 2 years	1	1	0.999^b^
**Pathology tumor stage, no. (%)**			0.793^a^
pTis Ta	8	12	
pT1	17	24	
pT2a	16	15	
pT2b	5	10	
pT3a	2	3	
pT3b	_	1	
pT4a	_	_	
pT4b	_	_	
**Pathologic lymph node status**			
Lymph node positive (pN+), no. (%)	2(4.2)	1(1.5)	0.574^b^
Lymph node yield, mean ± SD, *n*	18.9 ± 2.4	20.0 ± 2.0	0.638^c^
**Surgical margin, no. (%)**			0.999^b^
Positive	3 (6.3)	5 (7.7)	
Negative	45 (93.7)	60 (92.3)	

^a^Pearson chi-square test

^b^Fisher exact test

^c^Independent *t* test

### Urinary continence

When it comes to urinary continence, there was no difference between the ELRC and TLRC groups in terms of day or night continence at 12 months (Table 5). And the capacity of the pouch could be equal to a normal adult bladder capacity (≈ 400 ml) at 12 months after surgery (Table 5).

**Table 5:** Continence of 113 patients with bladder cancer treated by TLRC versus ELRC

Variables	Overall (*n* = 113)	ELRC group (*n* = 48)	TLRC group (*n* = 65)	*p* value
**Daytime incontinence at 12 months**				
0–1 pad/day, *n* (%)	103 (91.2)	44 (91.7)	59 (90.8)	1^a^
> 1 pad/day, *n* (%)	10 (8.8)	4 (8.3)	6 (9.2)	
**Nighttime incontinence at 12 months**				
0–1 pad/day, *n* (%)	97 (85.8)	42 (87.5)	55 (84.6)	0.788^a^
>1 pad/day, *n* (%)	16 (14.2)	6 (12.5)	10 (15.4)	
Neobladder capacity at 12 months, mean ± SD (range), ml	397.1 ± 80.5 (360–485)	390.9 ± 77.7 (370–485)	401.7 ± 94.8 (360–470)	0.695^c^

^a^Pearson chi-square test

^b^Fisher exact test

^c^Independent *t* test

## Discussion

To our knowledge, this is the first study assessing the impact of ELRC with ICUD on early postoperative recovery and complications. Transperitoneal approach is mostly used in radical cystectomy. However, complications following TLRC including ileus, urine leakage, and bowel fistula can complicate and prolong patients’ recovery. Due to absence of peritoneum and abandonment of natural compartmentalization between urinary and gastrointestinal systems, the transperitoneal approach resulted in high risks of inflammatory reactions or small serosa lesions which later lead to adhesions [13]. These adhesions often caused paralytic ileus, bloating, or constipation. The incidence of these gastrointestinal complications in TLRC was as high as 22% in recent studies [14–16]. Over the past decade, an increasing number of surgeons have voiced the significance of preserving the integrity of the peritoneum. Roth B et al. first revealed that readaptation of peritoneal layer after pelvic lymphadenectomy (PLND) and cystectomy could result in significantly less postoperative pain, faster recovery of bowel function, and fewer complications in the early postoperative period [13]. Moreover, Kulkari et al. proposed am extraperitoneal approach to minimize bowel injury, bowel adhesion, and injury in 1999 for radical cystectomy and bilateral pelvic lymphadenectomy [3].

As shown in literature, the final step of ELRC is to separate the bladder and peritoneum [17]. With early return of peristalsis, the incidence of postoperative ileus would be lower, mainly attributed to opening the peritoneum late and close the peritoneum promptly after establishing a neobladder [6]. According to previous reports, open peritoneal approach was superior to transperitoneal approach in decreasing gastrointestinal complications and improving bowel recovery especially for older people [6, 18]. In our study, in order to evaluate bowel recovery speed, we mainly referred to the time to flatus, the interval to tolerating solid food, LOS, and the incidence of postoperative ileus. The bowel recovery results appeared to favor the ELRC group. The ELRC group showed significantly shorter interval to flatus (35.5 ± 9.7 vs 42.7 ± 10.8 h, *p* < 0.001), shorter days to solid food (4.8 ± 1.3 vs 6.0 ± 1.7 days, *p* < 0.01), shorter LOS (12.7 ± 2.0 vs 14.0 ± 2.7 days, *p* < 0.01), and lower incidence of postoperative gastrointestinal complications (2.1% vs 14.9%, *p* < 0.05), compared to the TLRC group. These results were consistent with the concept of utilizing laparoscopic surgery in extraperitoneal radical cystectomy to reduce complications, as proposed by Zhao et al. [19]. As shown in numerous studies, laparoscopic or robotic assisted radical cystectomy with ICUD has been proven feasible and safe relative to conventional extracorporeal urinary diversion (ECUD) or open radical cystectomy (ORC) [5, 20–22]. In general, our technique is a combination of ELRC and ICUD which may be an alternative for surgeons in centers without robotic systems.

Late complications were mostly urinary diversion related, such as bladder stone, uretero-ileal anastomotic stenosis, and vesical-urethral anastomotic stricture. With regard to these complications, no significant difference was identified in our follow-up. The overall complication rate was 27.1% vs 33.8% in the ELRC group vs TLRC group (*p* = 0.538). In clinical practice, urethral sparing and urethral sphincter sparing are commonly performed in both extraperitoneal or transperitoneal approaches [23]. This could possibly explain that in the long-time follow-up, there was no significant difference between the two groups in terms of urinary continence or neobladder related late complications [6, 24].

We also noticed some complication rates were higher than recent reports like uretero-ileal anastomotic stenosis rate (overall rate = 9.7) and incidence of pouch stones (overall rate = 5.3). Actually, we are working to improve surgery, reduce surgical complications and ease patient suffering. Frankly speaking, the high incidence of bladder stones is related to two factors. One side was using of endo-staple (metallic) in the suturing of neobladder. On the other hand, water-drinking habits in older patients who preferred strong tea in China could accelerate bladder stone formation. Due to uretero-ileal anastomotic stenosis, we have used the reflux suturing technique (Wallace technique) in uretero-ileal anastomosis to replace the anti-reflux technique from 2020. Now the relevant follow-up data are being collated, and we hope the final result was better than anti-reflux technique.

Regarding extraperitoneal approach and laparoscopic surgery, there has been a concern about oncological safety [25, 26]. In 2013, Zhu et al. stated that only tumors ≤ T2 stages were suitable for extraperitoneal approach, while patients with > T2 stage and positive lymph nodes were not suitable candidates for extraperitoneal approach [25]. Concerns mostly come from possible positive surgical margin (PSM) because of peritoneal preservation, which may increase the risk of metastatic progression and cancer-specific mortality. Furthermore, we compared the overall PSM rate with previous reports. Our overall PSM rate was 7%. In a USA cohort, the overall RARC PSM rate was 6%, and in patients with prostate surgery history it even was much higher to 14% [27]. Also, in a large multicenter report about PSM in radical cystectomy pointed that the PSM rate was 10.2% [28]. PSM could be related to many factors, including prostate surgery history, pelvic adhesion, or tumor metastasis which could not be identified before surgery [27]. In multiple studies, laparoscopic radical cystectomy and open technique showed no significant difference in PSM [29–31].

Recently, several studies in the same period have shown that there was no oncological difference between the extraperitoneal approach and the transperitoneal approach. With a mean follow-up up to 10 years, the local recurrence and distant metastasis results were similar even with higher tumor stage in the extraperitoneal group [6, 26]. Such results were identified by Mihai et al. in their long-term results of a prospective randomized trial assessing the impact of re-adaptation of the dorsolateral peritoneal layer, which was also a technique to keep the integrity of peritoneum [32]. Regarding oncological outcomes, patients with ≤ T2 stage were recommended to receive ELRC. And in our follow-up, we found no difference between ELRC and TLRC regarding the postoperative pathologic outcomes, including positive surgical margin rate and lymph node yield. In the follow-up, local recurrence and distant metastasis were comparable between the two groups.

On the other hand, for patients with higher tumor stage (> pT2N0M0), NAC could make ELRC available. Charles C et al. were able to show that downstaging rate was 52.2% for ddMVAC and the complete response (pT0N0) was even up to 41.3% [33]. Francesco et al. also reported an encouraging outcome with downstaging to non-muscle-invasive disease (< pT2N0M0) in 55% patients [34], which could really reduce the difficulty of RC surgery and offer opportunities for patients to receive ELRC. In our study, patients (> T2N0M0) received NAC before radical cystectomy, which really gave them another choice besides traditional TLRC.

Another major concern was the high incidence of lymphocele after extraperitoneal approach since peritoneum over the iliac vessels might block lymph drainage into the peritoneal cavity where the lymph fluid was reabsorbed [13]. Latest research showed that open peritoneal approaches had similar symptomatic lymphocele incidence for extraperitoneal and transperitoneal approaches [35]. In our study, no statistically different lymphocele results were observed between the two groups, and the overall lymphocele rate for both groups was relatively low. In the process of surgery, 3D laparoscopic technique made the surgical vision field clear. Second, polydioxanone sutures were feasible to keep the flow of lymph drainage. It is important to keep the drainage patent whether lymphocele has developed or not.

This study has some certain limitations. First, the study was a retrospective study of non-randomized patients, and selection bias may influence outcomes. Second, the sample size was small. Third, the follow-up was not long enough to decide longer term complications and oncological outcomes.

## Conclusions

Surgical technique of ELRC with ICUD can achieve the established oncologic criteria of TLRC, and such technique can improve perioperative and early postoperative outcomes. However, long-term follow-up is needed for its further confirmation. In the next step, prospective randomized trials are essential to prove the real advantages of ELRC and ICUD.

## Data Availability

Data is available on request from the corresponding author by email: zhangyingamu@qq.com or biliangkuan118@yeah.net.

## Abbreviations

MIBC: Muscle invasive bladder cancer
ASA: American Society of Anesthesiologists
LRC: Laparoscopic radical cystectomy
PLND: Pelvic lymphadenectomy
BMI: Body mass index
TUR-BT: Transurethral bladder tumor resection
UTI: Urinary tract infection
RARC: Robot-assisted radical cystectomy
HRQOL: Health-related quality of life
NS: Nerve sparing
UD: Urinary diversion
ECUD: Extracorporeal urinary diversion
ICUD: Intracorporeal urinary diversion
LOS: Length of hospital stay
EBL: Estimated blood loss
ICU: Intensive care unit
AKI: Acute renal insufficiency
ERRC: Extraperitoneal robotic radical cystectomy
NAC: New adjuvant chemotherapy
PSM: Positive surgical margin
